# Educational inequality in physician-diagnosed hypertension widened and persisted among women from 1999 to 2014 in Hong Kong

**DOI:** 10.1038/s41598-019-50760-6

**Published:** 2019-10-07

**Authors:** Gary K. K. Chung, Francisco T. T. Lai, Eng-Kiong Yeoh, Roger Y. Chung

**Affiliations:** The Jockey Club School of Public Health and Primary Care, Faculty of Medicine, The Chinese University of Hong Kong, Shatin, New Territories, Hong Kong, China

**Keywords:** Hypertension, Epidemiology

## Abstract

Gender differences in the trend of educational inequality in hypertension have been observed especially in the Asian populations, indicating the increasing importance of education as a social determinant of hypertension among women. This study examined the gender-specific trends of educational inequality in physician-diagnosed hypertension in Hong Kong between 1999 and 2014. Based on a series of eight government-led territory-wide household surveys conducted between 1999 and 2014, 97,481 community-dwelling Hong Kong Chinese adults aged 45 or above were analysed. The extent and trend of gender-specific educational inequality in self-reported physician-diagnosed hypertension were estimated by regression-based Relative Index of Inequality and age-standardised Slope Index of Inequality. Over the study period, age-standardised prevalence of self-reported hypertension increased in both genders, with the greatest prevalence among the least educated women. Educational inequalities in hypertension significantly widened in female from 1999 to 2009 and persisted thereafter; nonetheless, the respective inequality was negligible in male. Further adjustment for household income did not attenuate the observed inequality. To conclude, a widened and then persistent discrepancy in hypertension across education levels was observed among women, but not among men, in Hong Kong. The gender perspective should be carefully considered when designing hypertension prevention strategies and related health policies.

## Introduction

Hypertension has long been recognized as a dominant preventable risk factor of numerous non-communicable diseases, especially cardiovascular and cerebrovascular diseases^1^, and also as the single leading cause of global disease burden^2^. While addressing raised blood pressure is the key to reducing premature non-communicable diseases mortality^3^, the disproportionate risk of hypertension across social groups remains a long-standing global challenge in hypertension prevention and control.

Education has long been considered the most important socioeconomic indicator of hypertension^4^. Apart from being a more upstream indicator of future employment and income, education uniquely reflects the cognitive functioning of an individual to respond to health information, and brings about higher efficiency as well as capacity in achieving health^5^. For women, education is a particularly important opportunity for empowerment, which helps them to gain social status and control over their lives especially in societies under strong gender biases^6^. The phenomenon of educational inequality in hypertension has a new dimension to it given the rapid dissemination of increasingly overwhelming health information via digital media since the turn of the century^7^. Despite the potential in improving population health, differential diffusion of health information could exacerbate social inequalities in health due to digital divides in terms of knowledge and information-seeking behaviours across education groups^8^. As a result, education is gaining importance as a social determinant of hypertension over the recent decades, and therefore the discrepancy in hypertension across education levels is expected to have widened.

While widening or persistent trends of educational inequality in hypertension and other cardiovascular risk factors have been well-documented in the Western populations over the past decades^9–11^, less relevant studies could be found in Asian settings for comparison. A few studies showed widening socioeconomic inequalities in nutrient intakes and obesity in women in South Korea^12,13^, and reported persistent negative associations with hypertension only in female since the 1990s in rapidly developing China^14^. Nonetheless, no studies in developed Asian setting, to our best knowledge, have investigated the trends of educational inequality in hypertension over the past two decades.

In the present study, we examined the gender-specific trends of educational inequality, in both relative and absolute terms, in hypertension between 1999 and 2014 in Hong Kong. Specifically, it is hypothesized that the inequality in hypertension across education levels would have widened over time, and more apparently among women.

## Methods

### Data collection and study population

This study was of a serial cross-sectional study design using population-representative data from eight territory-wide cross-sectional household surveys, the Thematic Household Survey (THS) commissioned by the Census and Statistics Department (C&SD) of the Government of the Hong Kong Special Administrative Region, in 1999, 2001, 2002, 2005, 2008, 2009, 2011 and 2014 for the present study. This series of eight surveys collected health-related data, including health status, healthcare utilization and hospitalization, medical benefits and insurance, sociodemographic and socioeconomic characteristics^15^.

The target population of THS covers the major population in Hong Kong; i.e., all land-based non-institutional Hong Kong Resident Population, excluding hotel transients, board vessel residents and foreign live-in domestic helpers^15^. It is based on a sample of living quarters selected from all permanent quarters and quarters in segments which are for residential and partially residential purposes in Hong Kong. The sampling frame of the survey consisted of all addresses of permanent quarters in built-up areas and records of area segments in non-built-up areas. The use of area segments was necessary for ensuring confidentiality as these areas do not have clear addresses and thus cannot easily be identified. Face-to-face household interviews were administered. All surveys covered at least 95% of the Hong Kong Resident Population at their corresponding periods with response rates between 75% and 79%^15^. In the present study, a total sample of 97,481 subjects aged 45 years or above was included to assess the trends of educational inequality in hypertension in Hong Kong from 1999 to 2014.

### Ethical statement

No ethical approval and written consent are required as we conducted secondary data analysis using eight THS datasets conducted by the C&SD of the Hong Kong Government. In the conduct of all survey exercise, the C&SD complies with the Declaration of Professional Ethics of the International Statistical Institute.

### Dependent variable

The question related to the dependent variable in surveys conducted in 1999, 2001, 2002 and 2005 was “do you have any diseases that require long-term follow-up?”, and the respondents with positive response were then asked whether they had hypertension. The respective question in surveys conducted in 2008, 2009, 2011 and 2014 was slightly modified into “have you ever been told by a western medicine practitioner that you had the following chronic health conditions?”, with hypertension as a response item. Self-reported status of hypertension was used as the binary dependent variable.

### Independent variables

Socioeconomic factors including educational attainment and household income were collected for the present study. The highest levels of education completed by the respondents were obtained and regrouped into “Below primary level”, “Primary level”, “Secondary level”, and “Tertiary level”. Data on monthly household income, in Hong Kong dollars (1USD = 7.8HKD), were collected by self-reported question with ordinal options of 12 income groups ranging from “below $4,000” to “$50,000 or above”, which were further regrouped into “$9,999 or below”, “$10,000–24,999”, “$25,000–49,999”, and “$50,000 or above” for descriptive analyses. Apart from education and household income, sociodemographic indicators including gender, age (classified into five groups from “45–49 years” to “65 years or above”), marital status (classified into “married” including cohabitation, and “non-married” for those never married, divorced, separated or widowed), and household size (classified into five groups from “single household” to “five members or above”) were also collected for standardisation or confounding control.

### Statistical methods

Sample characteristics across survey years and stratified by gender were presented as frequencies with column percentages. Prevalence of hypertension across education and household income levels were age-standardised with reference to the overall and gender-specific age structures of the population in mid-2016 in Hong Kong. In case no respondents were observed in an age-specific education or income group, the corresponding age-standardized prevalence could not be calculated and therefore would not be provided.

#### Multivariable analyses

Relative Index of Inequality and age-standardised Slope Index of Inequality: To quantify the extent of educational inequality in hypertension, in both relative and absolute terms, the Relative Index of Inequality (RII) and age-standardised Slope Index of Inequality (SII) were derived as two major summary measures^16^. Education and household income levels of respondents were ranked within populations of the corresponding year, and were separately assigned a fractional rank score scaled from 0 (highest socioeconomic status) to 1 (lowest socioeconomic status). To account for gender-specific variations of socioeconomic distributions, the fractional rank scores were also separately derived for male and female respondents. Then, the fractional rank scores were used as independent variables in generalised linear regression models for binomial dependent variables^16^. RIIs were assessed by the coefficients of fractional rank scores using a logarithmic link function, while the age-standardised SIIs were calculated by the age-specific coefficients using an identity link function, with adjustments for age groups, gender (not adjusted in gender-specific analyses), marital status and household size. Age-standardisation is important for a meaningful comparison of SIIs across populations with changing age structures over time. Regarding interpretations, RIIs above 1 and SIIs above 0 represent inequality in favour of respondents with higher socioeconomic statuses. The two inequality measures should be regarded as the expected relative and excess risk between two hypothetical extremes of the socioeconomic rank scale. Model-robust approaches were used to obtain standard errors to construct 95% confidence intervals (CI) and *p*-values^16^.

All data analyses were conducted using statistical software R 3.4.0 and *p*-values < 0.05 were regarded to be statistically significant.

## Results

### Descriptive statistics

Basic characteristics of the 97,481 adults aged 45 years or above sampled between 1999 and 2014 are presented in Table 1. The crude overall prevalence of self-reported hypertension approximately doubled from 1999 to 2014. Similar patterns were observed in both genders (Supplementary Tables S1 and S2).

**Table 1:** Basic characteristics of all respondents (N = 97,481).

	1999		2001		2002		2005		2008		2009		2011		2014		Total	
	N	(Column %)	N	(Column %)	N	(Column %)	N	(Column %)	N	(Column %)	N	(Column %)	N	(Column %)	N	(Column %)	N	(Column %)
**All**	10,545		11,228		10,672		12,025		12,651		13,003		13,457		13,900		97,481	
*Age*																		
45–49	2,441	(23.1%)	2,571	(22.9%)	2,443	(22.9%)	2,842	(23.6%)	2,730	(21.6%)	2,670	(20.5%)	2,617	(19.4%)	2,274	(16.4%)	20,588	(21.1%)
50–54	1,979	(18.8%)	2,285	(20.4%)	2,063	(19.3%)	2,504	(20.8%)	2,595	(20.5%)	2,693	(20.7%)	2,700	(20.1%)	2,742	(19.7%)	19,561	(20.1%)
55–59	1,155	(11.0%)	1,252	(11.2%)	1,296	(12.1%)	1,749	(14.5%)	1,899	(15.0%)	1,938	(14.9%)	2,176	(16.2%)	2,368	(17.0%)	13,833	(14.2%)
60–64	1,359	(12.9%)	1,241	(11.1%)	1,167	(10.9%)	1,190	(9.9%)	1,385	(10.9%)	1,685	(13.0%)	1,760	(13.1%)	2,004	(14.4%)	11,791	(12.1%)
65 or above	3,611	(34.2%)	3,879	(34.5%)	3,703	(34.7%)	3,740	(31.1%)	4,042	(32.0%)	4,017	(30.9%)	4,204	(31.2%)	4,512	(32.5%)	31,708	(32.5%)
*Gender*																		
Female	5,251	(49.8%)	5,603	(49.9%)	5,355	(50.2%)	5,994	(49.8%)	6,332	(50.1%)	6,667	(51.3%)	7,022	(52.2%)	7,277	(52.4%)	49,501	(50.8%)
Male	5,294	(50.2%)	5,625	(50.1%)	5,317	(49.8%)	6,031	(50.2%)	6,319	(49.9%)	6,336	(48.7%)	6,435	(47.8%)	6,623	(47.6%)	47,980	(49.2%)
*Marital status*																		
Married	8,237	(78.1%)	9,239	(82.3%)	8,233	(77.1%)	9,459	(78.7%)	9,613	(76.0%)	9,881	(76.0%)	10,067	(74.8%)	10,374	(74.6%)	75,103	(77.0%)
Non-married	2,283	(21.7%)	1,989	(17.7%)	2,432	(22.8%)	2,566	(21.3%)	3,038	(24.0%)	3,122	(24.0%)	3,390	(25.2%)	3,526	(25.4%)	22,346	(22.9%)
Missing	25	(0.2%)	0	(0.0%)	7	(0.1%)	0	(0.0%)	0	(0.0%)	0	(0.0%)	0	(0.0%)	0	(0.0%)	32	(0.0%)
*Household size*																		
1	774	(7.3%)	655	(5.8%)	1,069	(10.0%)	897	(7.5%)	1,110	(8.8%)	1,177	(9.1%)	1,233	(9.2%)	1,237	(8.9%)	8,152	(8.4%)
2	1,890	(17.9%)	1,941	(17.3%)	2,411	(22.6%)	2,587	(21.5%)	2,863	(22.6%)	3,123	(24.0%)	3,125	(23.2%)	3,512	(25.3%)	21,452	(22.0%)
3	2,166	(20.5%)	2,498	(22.2%)	2,572	(24.1%)	3,191	(26.5%)	3,338	(26.4%)	3,474	(26.7%)	3,643	(27.1%)	3,750	(27.0%)	24,632	(25.3%)
4	2,871	(27.2%)	3,413	(30.4%)	2,640	(24.7%)	3,229	(26.9%)	3,350	(26.5%)	3,354	(25.8%)	3,516	(26.1%)	3,501	(25.2%)	25,874	(26.5%)
5 or above	2,844	(27.0%)	2,721	(24.2%)	1,980	(18.6%)	2,121	(17.6%)	1,990	(15.7%)	1,875	(14.4%)	1,940	(14.4%)	1,900	(13.7%)	17,371	(17.8%)
*Education*																		
Below primary level	2,486	(23.6%)	2,226	(19.8%)	2,341	(21.9%)	1,895	(15.8%)	1,634	(12.9%)	1,435	(11.0%)	1,448	(10.8%)	1,365	(9.8%)	14,830	(15.2%)
Primary level	3,951	(37.5%)	4,386	(39.1%)	3,872	(36.3%)	4,066	(33.8%)	4,403	(34.8%)	4,324	(33.3%)	4,132	(30.7%)	4,198	(30.2%)	33,332	(34.2%)
Secondary level	3,325	(31.5%)	3,805	(33.9%)	3,629	(34.0%)	5,021	(41.8%)	5,537	(43.8%)	6,048	(46.5%)	6,454	(48.0%)	6,736	(48.5%)	40,555	(41.6%)
Tertiary level	760	(7.2%)	811	(7.2%)	817	(7.7%)	1,043	(8.7%)	1,077	(8.5%)	1,196	(9.2%)	1,423	(10.6%)	1,601	(11.5%)	8,728	(9.0%)
Missing	23	(0.2%)	0	(0.0%)	13	(0.1%)	0	(0.0%)	0	(0.0%)	0	(0.0%)	0	(0.0%)	0	(0.0%)	36	(0.0%)
*Household income (HKD)*																		
$9999 or less	2,906	(27.6%)	3,124	(27.8%)	3,414	(32.0%)	2,927	(24.3%)	3,572	(28.2%)	3,837	(29.5%)	3,182	(23.6%)	2,790	(20.1%)	25,752	(26.4%)
$10000–24999	3,837	(36.4%)	4,140	(36.9%)	3,838	(36.0%)	4,914	(40.9%)	4,828	(38.2%)	5,018	(38.6%)	4,806	(35.7%)	4,626	(33.3%)	36,007	(36.9%)
$25000–49999	2,325	(22.0%)	2,829	(25.2%)	2,034	(19.1%)	3,054	(25.4%)	3,003	(23.7%)	2,928	(22.5%)	3,990	(29.6%)	4,454	(32.0%)	24,617	(25.3%)
$50000 or above	902	(8.6%)	1,135	(10.1%)	665	(6.2%)	831	(6.9%)	1,248	(9.9%)	1,220	(9.4%)	1,479	(11.0%)	2,030	(14.6%)	9,510	(9.8%)
Missing	575	(5.5%)	0	(0.0%)	721	(6.8%)	299	(2.5%)	0	(0.0%)	0	(0.0%)	0	(0.0%)	0	(0.0%)	1,595	(1.6%)
*Diabetes*																		
No	9,897	(93.9%)	10,433	(92.9%)	9,909	(92.9%)	11,158	(92.8%)	11,522	(91.1%)	11,794	(90.7%)	12,033	(89.4%)	12,479	(89.8%)	89,225	(91.5%)
Yes	648	(6.1%)	795	(7.1%)	763	(7.1%)	867	(7.2%)	1,129	(8.9%)	1,209	(9.3%)	1,424	(10.6%)	1,421	(10.2%)	8,256	(8.5%)

### Age-standardised prevalence of hypertension

**Figure 1:**
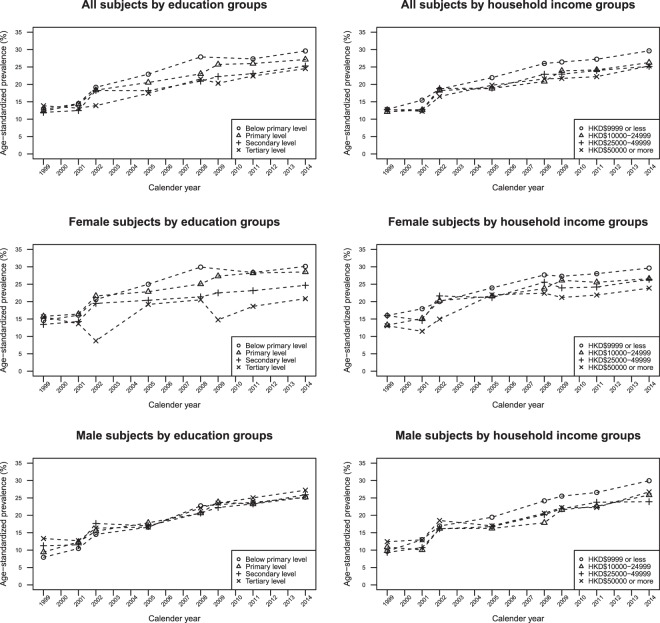
Trends of overall and gender-specific prevalence of hypertension by education and household income groups between 1999 and 2014.

Figure 1 presents the total and gender-specific age-standardised prevalence of hypertension across education levels and household income levels. A surge in prevalence was observed in both genders. The prevalence of hypertension in all education and household income groups did not deviate much in earlier survey years. However, the extent of surge in female differed across education levels, but not across household income levels, until 2009 with a more apparent increase in less educated women. The prevalence among women remained stable across all education groups thereafter. Nonetheless, no differential increase either by education or household income levels was observed in male.

### Relative and absolute educational inequalities in hypertension

**Figure 2:**
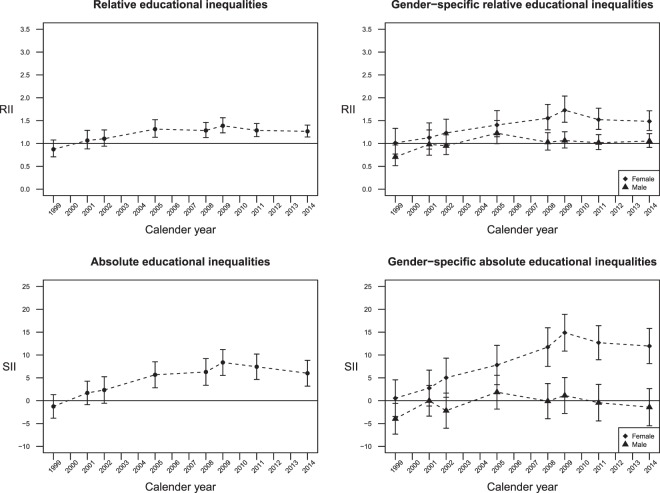
Trends of overall and gender-specific relative and absolute educational inequalities in hypertension between 1999 and 2014.

Relative and absolute educational inequalities across survey years are depicted in Fig. 2. Increasing RIIs and SIIs were observed in the overall sample; however, the trends were mostly driven by the widening inequality in hypertension in female, since there was no significant inequality in male except for a positive association in 1999. On the other hand, RIIs and SIIs in female increased from 1.01 (95% CI = 0.77–1.33) to 1.48 (95% CI = 1.28–1.72), and from 0.55% (95% CI = −3.46–4.57%) to 11.96% (95% CI = 8.11%–15.80%), respectively, between 1999 and 2014, with a peak in 2009 (RII = 1.73, 95% CI = 1.46–2.04; SII = 14.88%; 95% CI = 10.85–18.91%) (Supplementary Table S3). After further adjustments for household income level, the extent of educational inequality did not significantly attenuate, and the overall patterns remained unchanged (Supplementary Table S4).

## Discussion

This is the first study to examine gender-specific trends of educational inequality in physician-diagnosed hypertension over the recent two decades using a series of population-representative surveys in Hong Kong. In addition to the overall rising trend in both genders, our study revealed that the surge in hypertension among the less educated women was substantially higher than that for their more educated counterparts between 1999 and 2009, leading to a widening educational inequality in hypertension, in both relative and absolute terms, in female during the period. Afterwards, the surge levelled off among the less educated women and became similar across education levels.; therefore, the educational inequality in hypertension among women persisted after 2009. Nonetheless, negligible educational inequality was found in male. Also, the observed educational inequality in hypertension was independent of household income level.

The rising trend in physician-diagnosed hypertension in Hong Kong could have partially been driven by the heightened health awareness and detection over time^15,17^, along with a growing number of local health education programs in the community as well as an earlier and more proactive detection in the primary care setting^18–20^. While the increasingly prevalent obesity, as a major risk factor to most cardiovascular events, may have contributed to the rise in hypertension, recent local research on validation of self-reported hypertension against the objective clinical data showed an improved case reporting as the level of sensitivity increased between 2003/04 and 2014/15 (Chung GK, 2019, unpublished data), which led to a rise in self-reported prevalence. Nonetheless, despite the potential overestimation of the extent of prevalence surge using self-reported data, the same local study showed no educational difference in the levels of sensitivity and positive predictive values for self-reported hypertension in both genders over the past decade (Chung GK, 2019, unpublished data). Therefore, the validity of our observed gender-specific trends of educational inequality in hypertension is ensured.

Our finding supports previous Asian studies showing gender-specific trends of educational inequality in hypertension and related cardiovascular risk factors^12–14^. The more apparent widening trend among women is consistent with the stronger educational gradient in incident hypertension among women relative to men in developed Asia^20^, and could possibly be attributed to the greater educational inequality in obesity among women generally observed in developed world regions^21^. Nonetheless, we speculate that the emergence of digital media since the turn of the century and its resulted digital divides across social groups could most plausibly explain our observed gender-specific widening trends between 1999 and 2009. During the early phase of the introduction of digital media in the early 2000s, access to the Internet and online health information were substantially lower among the socially disadvantaged groups^8^. Even in later phases with reduced barriers to access to the Internet due to increasing coverage of the Internet and use of smart phones, discrepancies in knowledge related to the utilization of the Internet, attitude and behaviours towards health information searching and capabilities to responding to the online information still exist across genders and socioeconomic levels^8^. Previous studies revealed that women and the more educated have better health awareness and they tend to seek health information more frequently via various communication channels^22,23^. In addition, highly educated women are generally more knowledgeable and capable of coping with the surge of health information, while the less educated women, who tend to respond maladaptively to similar information, become more likely to experience negative emotions and thus reduced behavioral intentions to preventive behaviors such as exercise and fruit and vegetable consumption^24,25^. Since prevention of hypertension depends largely on health information and lifestyle practice, digital divides could have a critical impact on the widening educational inequality in hypertension during the emergence of digital media^8^. Subsequently, digital divides are expected to have diminished as the use of the Internet and digital media has been increasingly incorporated into our daily life regardless of socioeconomic background, which may partly explain our observed persistent inequality after 2009. Moreover, another possible reason for the persistent trend could be the strengthened health promotion targeted to the socially disadvantaged groups in the community over the past decade^20^, leading to the levelling-off of prevalence surge among the less educated women.

While the rising income inequality in Hong Kong^26^ could be another driver of our observed widened and then persistent educational inequality in hypertension, our study showed negligible attenuation of educational inequality by household income level. This finding supports previous studies that education is a more important socioeconomic indicator than income in terms of health behaviours, coping strategies and health information seeking behaviours^23,24,27^. Nonetheless, recent research showed that deprivation of material and social necessities is associated with obesity and self-rated health above and beyond the impact of education and income^28,29^, which may play a part in explaining the observed educational inequality.

The particularly stronger gender differences in educational inequalities in hypertension and related cardiovascular risk factors in Asia^11–14^ could partially be attributed to the greater gender biases under Asian cultural orientation^30^. Although Hong Kong offers relatively equal employment opportunities between genders^30^, traditional gender roles still prevail in wider society due to the patriarchal nature among Chinese populations^31^. In Asian societies with strong gender biases, socially disadvantaged women share lower decision making power and control over basic necessities and opportunities for health achievement^6^. The gender biases do not only operate at societal level but also at household level since women of low social status generally have lower access to household resources in a family^32^. This situation in Hong Kong might be even worse given the recent influx of female migrants from the Mainland China who tend to be less educated and, meanwhile, face greater discrimination^33^.

In light of the widened and then persistent educational inequality in hypertension in female, the less educated women could serve as a potential target segment for tailor-made interventions in Hong Kong. Since the observed educational inequality is independent of household income level, conventional endowment policies may not be adequate. Instead, designing health messages appropriate to the literacy level of the less educated women may be more conducive to favourable behavioural changes for reducing the prevalence of and inequality in hypertension. The experience in Hong Kong may also inform gender-specific high-risk prevention strategies in other emerging Asian economies so as to avoid the potential rising inequalities along with rapid economic development^34^.

There are several potential limitations in our study. Firstly, underestimation of prevalence is common as self-reported hypertension was adopted; nonetheless, previous studies supported reasonable validity of using self-reported information to assess physician-diagnosed hypertension in the community^35^, and showed no difference in underreporting across education levels (Chung GK, 2019, unpublished data). Secondly, the age-standardized prevalence for respondents with “Below primary level” of education in 2009 was not provided in Fig. 1 because there were no respondents with less than primary education in the 45–49 age group in 2009, and therefore we could not derive its age-specific rate for the calculation of the age-standardized prevalence; nonetheless, this would not affect the results of the subsequent multiple logistic regression analyses. Thirdly, residual confounding such as medical history and differences in genetic composition may exist despite adjustments for age, marital status, household size, and additionally for household income. It is, however, worth noting that nutritional outcomes like obesity should not be considered as confounders as they are highly socially patterned and thus may serve as mediators in the association between education and hypertension. Lastly, since SIIs were age-standardised but RIIs were not, directions of SIIs may differ from that of RIIs in the same year in case the association is close to null. Therefore, the two measures should be compared with caution.

To conclude, a widened and then persistent discrepancy across education levels is apparent among women in Hong Kong. Less educated, but not necessarily income-poor, women are expected to have greater cardiovascular disease burden in the coming decade, and hence warrant special attention in disease prevention and management. Policy makers should be aware of the gender difference when designing hypertension prevention strategies and related health policies to reduce the educational inequality.

## Supplementary information

Supplementary Tables S1–4

## Data Availability

The data that support the findings of this study are available from C&SD of the Hong Kong Government but restrictions apply to the availability of these data, which were used under license for the current study, and so are not publicly available. Data are available from the authors upon reasonable request and with permission of C&SD of the Hong Kong Government.
