# Supplementary material for: Educational inequality in physician-diagnosed hypertension widened and persisted among women from 1999 to 2014 in Hong Kong
Source: Sci Rep. 2019 Oct 7;9:14361. doi: 10.1038/s41598-019-50760-6 (PMC6779914; doi:10.1038/s41598-019-50760-6)
Supplement: Supplementary file 1 — Supplementary Tables S1–4 [file 41598_2019_50760_MOESM1_ESM.docx]

**Educational inequality in physician-diagnosed hypertension widened and persisted among women from 1999 to 2014 in Hong Kong**

Running head: Trends of hypertension by social groups

Gary K.K. CHUNG^a^, Francisco T.T. LAI^a^, Eng-Kiong YEOH^a^, Roger Y. CHUNG^a^*

^a^ The Jockey Club School of Public Health and Primary Care, Faculty of Medicine, The Chinese University of Hong Kong, Shatin, New Territories, Hong Kong, China

*Corresponding Author

**Name:** Roger Yat-Nork CHUNG

**Phone:** +852 2252-8799

**Fax:** +852 2606-3791

**Email address:** rychung@cuhk.edu.hk

**Funding:** This research did not receive any specific grant from funding agencies in the public, commercial, or not-for-profit sectors.

| **Supplementary Table S1. Basic characteristics of female respondents (N=49,501)** | | | | | | | | | | | | | | | | | | | | | | | | | | | | |
| --- | --- | --- | --- | --- | --- | --- | --- | --- | --- | --- | --- | --- | --- | --- | --- | --- | --- | --- | --- | --- | --- | --- | --- | --- | --- | --- | --- | --- |
|  |  |  | 1999 | |  | 2001 | |  | 2002 | |  | 2005 | |  | 2008 | |  | 2009 | |  | 2011 | |  | 2014 | |  | Total | |
|  |  |  | N | (Column %) |  | N | (Column %) |  | N | (Column %) |  | N | (Column %) |  | N | (Column %) |  | N | (Column %) |  | N | (Column %) |  | N | (Column %) |  | N | (Column %) |
| **Female** | | | 5,251 |  |  | 5,603 |  |  | 5,355 |  |  | 5,994 |  |  | 6,332 |  |  | 6,667 |  |  | 7,022 |  |  | 7,277 |  |  | 49,501 |  |
|  | *Age* | |  |  |  |  |  |  |  |  |  |  |  |  |  |  |  |  |  |  |  |  |  |  |  |  |  |  |
|  |  | 45-49 | 1,208 | (23.0%) |  | 1,295 | (23.1%) |  | 1,242 | (23.2%) |  | 1,423 | (23.7%) |  | 1,360 | (21.5%) |  | 1,434 | (21.5%) |  | 1,455 | (20.7%) |  | 1,232 | (16.9%) |  | 10,649 | (21.5%) |
|  |  | 50-54 | 953 | (18.1%) |  | 1,096 | (19.6%) |  | 991 | (18.5%) |  | 1,276 | (21.3%) |  | 1,297 | (20.5%) |  | 1,326 | (19.9%) |  | 1,364 | (19.4%) |  | 1,430 | (19.7%) |  | 9,733 | (19.7%) |
|  |  | 55-59 | 503 | (9.6%) |  | 578 | (10.3%) |  | 640 | (12.0%) |  | 830 | (13.8%) |  | 929 | (14.7%) |  | 983 | (14.7%) |  | 1,073 | (15.3%) |  | 1,242 | (17.1%) |  | 6,778 | (13.7%) |
|  |  | 60-64 | 639 | (12.2%) |  | 594 | (10.6%) |  | 529 | (9.9%) |  | 562 | (9.4%) |  | 666 | (10.5%) |  | 846 | (12.7%) |  | 907 | (12.9%) |  | 1,031 | (14.2%) |  | 5,774 | (11.7%) |
|  |  | 65 or above | 1,948 | (37.1%) |  | 2,040 | (36.4%) |  | 1,953 | (36.5%) |  | 1,903 | (31.7%) |  | 2,080 | (32.8%) |  | 2,078 | (31.2%) |  | 2,223 | (31.7%) |  | 2,342 | (32.2%) |  | 16,567 | (33.5%) |
|  | *Marital status* | |  |  |  |  |  |  |  |  |  |  |  |  |  |  |  |  |  |  |  |  |  |  |  |  |  |  |
|  |  | Married | 3,616 | (68.9%) |  | 4,222 | (75.4%) |  | 3,655 | (68.3%) |  | 4,274 | (71.3%) |  | 4,308 | (68.0%) |  | 4,473 | (67.1%) |  | 4,617 | (65.8%) |  | 4,806 | (66.0%) |  | 33,971 | (68.6%) |
|  |  | Non-married | 1,624 | (30.9%) |  | 1,381 | (24.6%) |  | 1,694 | (31.6%) |  | 1,720 | (28.7%) |  | 2,024 | (32.0%) |  | 2,194 | (32.9%) |  | 2,405 | (34.2%) |  | 2,471 | (34.0%) |  | 15,513 | (31.3%) |
|  |  | Missing | 11 | (0.2%) |  | 0 | (0.0%) |  | 6 | (0.1%) |  | 0 | (0.0%) |  | 0 | (0.0%) |  | 0 | (0.0%) |  | 0 | (0.0%) |  | 0 | (0.0%) |  | 17 | (0.0%) |
|  | *Household size* | |  |  |  |  |  |  |  |  |  |  |  |  |  |  |  |  |  |  |  |  |  |  |  |  |  |  |
|  |  | 1 | 374 | (7.1%) |  | 324 | (5.8%) |  | 541 | (10.1%) |  | 448 | (7.5%) |  | 539 | (8.5%) |  | 643 | (9.6%) |  | 716 | (10.2%) |  | 702 | (9.6%) |  | 4,287 | (8.7%) |
|  |  | 2 | 1,024 | (19.5%) |  | 1,049 | (18.7%) |  | 1,282 | (23.9%) |  | 1,394 | (23.3%) |  | 1,562 | (24.7%) |  | 1,720 | (25.8%) |  | 1,735 | (24.7%) |  | 1,955 | (26.9%) |  | 11,721 | (23.7%) |
|  |  | 3 | 1,071 | (20.4%) |  | 1,288 | (23.0%) |  | 1,317 | (24.6%) |  | 1,582 | (26.4%) |  | 1,666 | (26.3%) |  | 1,775 | (26.6%) |  | 1,865 | (26.6%) |  | 1,903 | (26.2%) |  | 12,467 | (25.2%) |
|  |  | 4 | 1,338 | (25.5%) |  | 1,572 | (28.1%) |  | 1,226 | (22.9%) |  | 1,512 | (25.2%) |  | 1,574 | (24.9%) |  | 1,577 | (23.7%) |  | 1,697 | (24.2%) |  | 1,738 | (23.9%) |  | 12,234 | (24.7%) |
|  |  | 5 or above | 1,444 | (27.5%) |  | 1,370 | (24.5%) |  | 989 | (18.5%) |  | 1,058 | (17.7%) |  | 991 | (15.7%) |  | 952 | (14.3%) |  | 1,009 | (14.4%) |  | 979 | (13.5%) |  | 8,792 | (17.8%) |
|  | *Education* | |  |  |  |  |  |  |  |  |  |  |  |  |  |  |  |  |  |  |  |  |  |  |  |  |  |  |
|  |  | Below primary level | 1,792 | (34.1%) |  | 1,609 | (28.7%) |  | 1,633 | (30.5%) |  | 1,319 | (22.0%) |  | 1,172 | (18.5%) |  | 1,050 | (15.7%) |  | 1,066 | (15.2%) |  | 1,023 | (14.1%) |  | 10,664 | (21.5%) |
|  |  | Primary level | 1,881 | (35.8%) |  | 2,168 | (38.7%) |  | 1,917 | (35.8%) |  | 2,078 | (34.7%) |  | 2,309 | (36.5%) |  | 2,331 | (35.0%) |  | 2,268 | (32.3%) |  | 2,311 | (31.8%) |  | 17,263 | (34.9%) |
|  |  | Secondary level | 1,315 | (25.0%) |  | 1,534 | (27.4%) |  | 1,522 | (28.4%) |  | 2,257 | (37.7%) |  | 2,454 | (38.8%) |  | 2,812 | (42.2%) |  | 3,129 | (44.6%) |  | 3,275 | (45.0%) |  | 18,298 | (37.0%) |
|  |  | Tertiary level | 252 | (4.8%) |  | 292 | (5.2%) |  | 275 | (5.1%) |  | 340 | (5.7%) |  | 397 | (6.3%) |  | 474 | (7.1%) |  | 559 | (8.0%) |  | 668 | (9.2%) |  | 3,257 | (6.6%) |
|  |  | Missing | 11 | (0.2%) |  | 0 | (0.0%) |  | 8 | (0.1%) |  | 0 | (0.0%) |  | 0 | (0.0%) |  | 0 | (0.0%) |  | 0 | (0.0%) |  | 0 | (0.0%) |  | 19 | (0.0%) |
|  | *Household income (HKD)* | |  |  |  |  |  |  |  |  |  |  |  |  |  |  |  |  |  |  |  |  |  |  |  |  |  |  |
|  |  | $9999 or less | 1,451 | (27.6%) |  | 1,584 | (28.3%) |  | 1,718 | (32.1%) |  | 1,505 | (25.1%) |  | 1,844 | (29.1%) |  | 2,041 | (30.6%) |  | 1,772 | (25.2%) |  | 1,559 | (21.4%) |  | 13,474 | (27.2%) |
|  |  | $10000-24999 | 1,849 | (35.2%) |  | 1,995 | (35.6%) |  | 1,919 | (35.8%) |  | 2,394 | (39.9%) |  | 2,366 | (37.4%) |  | 2,520 | (37.8%) |  | 2,458 | (35.0%) |  | 2,393 | (32.9%) |  | 17,894 | (36.1%) |
|  |  | $25000-49999 | 1,181 | (22.5%) |  | 1,447 | (25.8%) |  | 1,033 | (19.3%) |  | 1,532 | (25.6%) |  | 1,508 | (23.8%) |  | 1,495 | (22.4%) |  | 2,052 | (29.2%) |  | 2,302 | (31.6%) |  | 12,550 | (25.4%) |
|  |  | $50000 or above | 458 | (8.7%) |  | 577 | (10.3%) |  | 318 | (5.9%) |  | 406 | (6.8%) |  | 614 | (9.7%) |  | 611 | (9.2%) |  | 740 | (10.5%) |  | 1,023 | (14.1%) |  | 4,747 | (9.6%) |
|  |  | Missing | 312 | (5.9%) |  | 0 | (0.0%) |  | 367 | (6.9%) |  | 157 | (2.6%) |  | 0 | (0.0%) |  | 0 | (0.0%) |  | 0 | (0.0%) |  | 0 | (0.0%) |  | 836 | (1.7%) |
|  | *Diabetes* | |  |  |  |  |  |  |  |  |  |  |  |  |  |  |  |  |  |  |  |  |  |  |  |  |  |  |
|  |  | No | 4,882 | (93.0%) |  | 5,163 | (92.1%) |  | 4,935 | (92.2%) |  | 5,530 | (92.3%) |  | 5,775 | (91.2%) |  | 6,062 | (90.9%) |  | 6,275 | (89.4%) |  | 6,578 | (90.4%) |  | 45,200 | (91.3%) |
|  |  | Yes | 369 | (7.0%) |  | 440 | (7.9%) |  | 420 | (7.8%) |  | 464 | (7.7%) |  | 557 | (8.8%) |  | 605 | (9.1%) |  | 747 | (10.6%)  ) |  | 699 | (9.6%) |  | 4,301 | (8.7%) |

| **Supplementary Table S2.** **Basic characteristics of male respondents (N=47,980)** | | | | | | | | | | | | | | | | | | | | | | | | | | | | |
| --- | --- | --- | --- | --- | --- | --- | --- | --- | --- | --- | --- | --- | --- | --- | --- | --- | --- | --- | --- | --- | --- | --- | --- | --- | --- | --- | --- | --- |
|  |  |  | 1999 | |  | 2001 | |  | 2002 | |  | 2005 | |  | 2008 | |  | 2009 | |  | 2011 | |  | 2014 | |  | Total | |
|  |  |  | N | (Column %) |  | N | (Column %) |  | N | (Column %) |  | N | (Column %) |  | N | (Column %) |  | N | (Column %) |  | N | (Column %) |  | N | (Column %) |  | N | (Column %) |
| **Male** | | | 5,294 |  |  | 5,625 |  |  | 5,317 |  |  | 6,031 |  |  | 6,319 |  |  | 6,336 |  |  | 6,435 |  |  | 6,623 |  |  | 47,980 |  |
|  | *Age* | |  |  |  |  |  |  |  |  |  |  |  |  |  |  |  |  |  |  |  |  |  |  |  |  |  |  |
|  |  | 45-49 | 1,233 | (23.3%) |  | 1,276 | (22.7%) |  | 1,201 | (22.6%) |  | 1,419 | (23.5%) |  | 1,370 | (21.7%) |  | 1,236 | (19.5%) |  | 1,162 | (18.1%) |  | 1,042 | (15.7%) |  | 9,939 | (20.7%) |
|  |  | 50-54 | 1,026 | (19.4%) |  | 1,189 | (21.1%) |  | 1,072 | (20.2%) |  | 1,228 | (20.4%) |  | 1,298 | (20.5%) |  | 1,367 | (21.6%) |  | 1,336 | (20.8%) |  | 1,312 | (19.8%) |  | 9,828 | (20.5%) |
|  |  | 55-59 | 652 | (12.3%) |  | 674 | (12.0%) |  | 656 | (12.3%) |  | 919 | (15.2%) |  | 970 | (15.4%) |  | 955 | (15.1%) |  | 1,103 | (17.1%) |  | 1,126 | (17.0%) |  | 7,055 | (14.7%) |
|  |  | 60-64 | 720 | (13.6%) |  | 647 | (11.5%) |  | 638 | (12.0%) |  | 628 | (10.4%) |  | 719 | (11.4%) |  | 839 | (13.2%) |  | 853 | (13.3%) |  | 973 | (14.7%) |  | 6,017 | (12.5%) |
|  |  | 65 or above | 1,663 | (31.4%) |  | 1,839 | (32.7%) |  | 1,750 | (32.9%) |  | 1,837 | (30.5%) |  | 1,962 | (31.0%) |  | 1,939 | (30.6%) |  | 1,981 | (30.8%) |  | 2,170 | (32.8%) |  | 15,141 | (31.6%) |
|  | *Marital status* | |  |  |  |  |  |  |  |  |  |  |  |  |  |  |  |  |  |  |  |  |  |  |  |  |  |  |
|  |  | Married | 4,621 | (87.3%) |  | 5,017 | (89.2%) |  | 4,578 | (86.1%) |  | 5,185 | (86.0%) |  | 5,305 | (84.0%) |  | 5,408 | (85.4%) |  | 5,450 | (84.7%) |  | 5,568 | (84.1%) |  | 41,132 | (85.7%) |
|  |  | Non-married | 659 | (12.4%) |  | 608 | (10.8%) |  | 738 | (13.9%) |  | 846 | (14.0%) |  | 1,014 | (16.0%) |  | 928 | (14.6%) |  | 985 | (15.3%) |  | 1,055 | (15.9%) |  | 6,833 | (14.2%) |
|  |  | Missing | 14 | (0.3%) |  | 0 | (0.0%) |  | 1 | (0.0%) |  | 0 | (0.0%) |  | 0 | (0.0%) |  | 0 | (0.0%) |  | 0 | (0.0%) |  | 0 | (0.0%) |  | 15 | (0.0%) |
|  | *Household size* | |  |  |  |  |  |  |  |  |  |  |  |  |  |  |  |  |  |  |  |  |  |  |  |  |  |  |
|  |  | 1 | 400 | (7.6%) |  | 331 | (5.9%) |  | 528 | (9.9%) |  | 449 | (7.4%) |  | 571 | (9.0%) |  | 534 | (8.4%) |  | 517 | (8.0%) |  | 535 | (8.1%) |  | 3,865 | (8.1%) |
|  |  | 2 | 866 | (16.4%) |  | 892 | (15.9%) |  | 1,129 | (21.2%) |  | 1,193 | (19.8%) |  | 1,301 | (20.6%) |  | 1,403 | (22.1%) |  | 1,390 | (21.6%) |  | 1,557 | (23.5%) |  | 9,731 | (20.3%) |
|  |  | 3 | 1,095 | (20.7%) |  | 1,210 | (21.5%) |  | 1,255 | (23.6%) |  | 1,609 | (26.7%) |  | 1,672 | (26.5%) |  | 1,699 | (26.8%) |  | 1,778 | (27.6%) |  | 1,847 | (27.9%) |  | 12,165 | (25.4%) |
|  |  | 4 | 1,533 | (29.0%) |  | 1,841 | (32.7%) |  | 1,414 | (26.6%) |  | 1,717 | (28.5%) |  | 1,776 | (28.1%) |  | 1,777 | (28.0%) |  | 1,819 | (28.3%) |  | 1,763 | (26.6%) |  | 13,640 | (28.4%) |
|  |  | 5 or above | 1,400 | (26.4%) |  | 1,351 | (24.0%) |  | 991 | (18.6%) |  | 1,063 | (17.6%) |  | 999 | (15.8%) |  | 923 | (14.6%) |  | 931 | (14.5%) |  | 921 | (13.9%) |  | 8,579 | (17.9%) |
|  | *Education* | |  |  |  |  |  |  |  |  |  |  |  |  |  |  |  |  |  |  |  |  |  |  |  |  |  |  |
|  |  | Below primary level | 694 | (13.1%) |  | 617 | (11.0%) |  | 708 | (13.3%) |  | 576 | (9.6%) |  | 462 | (7.3%) |  | 385 | (6.1%) |  | 382 | (5.9%) |  | 342 | (5.2%) |  | 4,166 | (8.7%) |
|  |  | Primary level | 2,070 | (39.1%) |  | 2,218 | (39.4%) |  | 1,955 | (36.8%) |  | 1,988 | (33.0%) |  | 2,094 | (33.1%) |  | 1,993 | (31.5%) |  | 1,864 | (29.0%) |  | 1,887 | (28.5%) |  | 16,069 | (33.5%) |
|  |  | Secondary level | 2,010 | (38.0%) |  | 2,271 | (40.4%) |  | 2,107 | (39.6%) |  | 2,764 | (45.8%) |  | 3,083 | (48.8%) |  | 3,236 | (51.1%) |  | 3,325 | (51.7%) |  | 3,461 | (52.3%) |  | 22,257 | (46.4%) |
|  |  | Tertiary level | 508 | (9.6%) |  | 519 | (9.2%) |  | 542 | (10.2%) |  | 703 | (11.7%) |  | 680 | (10.8%) |  | 722 | (11.4%) |  | 864 | (13.4%) |  | 933 | (14.1%) |  | 5,471 | (11.4%) |
|  |  | Missing | 12 | (0.2%) |  | 0 | (0.0%) |  | 5 | (0.1%) |  | 0 | (0.0%) |  | 0 | (0.0%) |  | 0 | (0.0%) |  | 0 | (0.0%) |  | 0 | (0.0%) |  | 17 | (0.0%) |
|  | *Household income (HKD)* | |  |  |  |  |  |  |  |  |  |  |  |  |  |  |  |  |  |  |  |  |  |  |  |  |  |  |
|  |  | $9999 or less | 1,455 | (27.5%) |  | 1,540 | (27.4%) |  | 1,696 | (31.9%) |  | 1,422 | (23.6%) |  | 1,728 | (27.3%) |  | 1,796 | (28.3%) |  | 1,410 | (21.9%) |  | 1,231 | (18.6%) |  | 12,278 | (25.6%) |
|  |  | $10000-24999 | 1,988 | (37.6%) |  | 2,145 | (38.1%) |  | 1,919 | (36.1%) |  | 2,520 | (41.8%) |  | 2,462 | (39.0%) |  | 2,498 | (39.4%) |  | 2,348 | (36.5%) |  | 2,233 | (33.7%) |  | 18,113 | (37.8%) |
|  |  | $25000-49999 | 1,144 | (21.6%) |  | 1,382 | (24.6%) |  | 1,001 | (18.8%) |  | 1,522 | (25.2%) |  | 1,495 | (23.7%) |  | 1,433 | (22.6%) |  | 1,938 | (30.1%) |  | 2,152 | (32.5%) |  | 12,067 | (25.2%) |
|  |  | $50000 or above | 444 | (8.4%) |  | 558 | (9.9%) |  | 347 | (6.5%) |  | 425 | (7.0%) |  | 634 | (10.0%) |  | 609 | (9.6%) |  | 739 | (11.5%) |  | 1,007 | (15.2%) |  | 4,763 | (9.9%) |
|  |  | Missing | 263 | (5.0%) |  | 0 | (0.0%) |  | 354 | (6.7%) |  | 142 | (2.4%) |  | 0 | (0.0%) |  | 0 | (0.0%) |  | 0 | (0.0%) |  | 0 | (0.0%) |  | 759 | (1.6%) |
|  | *Diabetes* | |  |  |  |  |  |  |  |  |  |  |  |  |  |  |  |  |  |  |  |  |  |  |  |  |  |  |
|  |  | No | 5,015 | (94.7%) |  | 5,270 | (93.7%) |  | 4,974 | (93.5%) |  | 5,628 | (93.3%) |  | 5,747 | (90.9%) |  | 5,732 | (90.5%) |  | 5,758 | (89.5%) |  | 5,901 | (89.1%) |  | 44,025 | (91.8%) |
|  |  | Yes | 279 | (5.3%) |  | 355 | (6.3%) |  | 343 | (6.5%) |  | 403 | (6.7%) |  | 572 | (9.1%) |  | 604 | (9.5%) |  | 677 | (10.5%) |  | 722 | (10.9%) |  | 3,955 | (8.2%) |

| **Supplementary Table S3.** **Relative and absolute educational inequalities in diabetes across years** | | | | | | |  | |  | | |  | | |  | | |  | |
| --- | --- | --- | --- | --- | --- | --- | --- | --- | --- | --- | --- | --- | --- | --- | --- | --- | --- | --- | --- |
|  |  |  | 1999 | 2001 | 2002 | 2005 | | 2008 | | | 2009 | | | 2011 | | | 2014 | | |
| All | |  |  |  |  |  | |  | | |  | | |  | | |  | | |
|  | RII (95% CI) ^a^ | | 1.38 (1.00-1.91)* | 1.02 (0.78-1.34) | 1.42 (1.06-1.90)* | | 1.61 (1.23-2.12)*** | | | 1.48 (1.17-1.86)*** | | | 1.67 (1.34-2.09)*** | | | 1.64 (1.35-2.00)*** | | | 1.59 (1.30-1.94)*** |
|  | SII (95% CI) ^a^ | | 2.26 (0.36-4.17)* | 0.68 (-1.32-2.68) | 3.10 (1.06-5.15)** | | 4.49 (2.56-6.41)*** | | | 4.21 (2.18-6.24)*** | | | 5.44 (3.37-7.50)*** | | | 5.57 (3.53-7.61)*** | | | 5.43 (3.50-7.35)*** |
| Female | | |  |  |  | |  | | |  | | |  | | |  | | |  |
|  | RII (95% CI) ^b^ | | 1.56 (0.99-2.46) | 1.38 (0.94-2.02) | 1.83 (1.21-2.75)** | | 1.87 (1.24-2.81)** | | | 2.23 (1.57-3.19)*** | | | 2.29 (1.62-3.24)*** | | | 2.44 (1.83-3.24)*** | | | 1.80 (1.33-2.42)*** |
|  | SII (95% CI) ^b^ | | 2.99 (0.15-5.83)* | 3.25 (0.32-6.18)* | 5.29 (2.44-8.14)*** | | 4.99 (2.07-7.90)*** | | | 7.39 (4.61-10.18)*** | | | 7.63 (4.73-10.53)*** | | | 9.21 (6.47-11.96)*** | | | 6.26 (3.73-8.79)*** |
| Male | | |  |  |  | |  | | |  | | |  | | |  | | |  |
|  | RII (95% CI) ^b^ | | 1.30 (0.83-2.03) | 0.74 (0.51-1.08) | 1.04 (0.69-1.55) | | 1.41 (0.98-2.02) | | | 1.02 (0.76-1.39) | | | 1.25 (0.93-1.67) | | | 1.09 (0.83-1.43) | | | 1.40 (1.07-1.82)* |
|  | SII (95% CI) ^b^ | | 1.55 (-0.94-4.05) | -1.94 (-4.60-0.72) | 0.19 (-2.59-2.96) | | 2.54 (0.03-5.06)* | | | 0.55 (-2.42-3.52) | | | 2.26 (-0.67-5.19) | | | 1.19 (-1.85-4.23) | | | 3.56 (0.65-6.47)* |
| ^a^ Age group, gender, marital status, household size, education fractional rank score, and household income fractional rank score were included | | | | | | | | | | | | | | | | | | | |
| ^b^ Age group, marital status, household size, education fractional rank score, and household income fractional rank score were included | | | | | | | | | | | | | | | | | | | |

| **Supplementary Table S4.** **Relative and absolute educational inequalities in diabetes across years after further adjustments for household income** | | | | | | |  | |  | | |  |
| --- | --- | --- | --- | --- | --- | --- | --- | --- | --- | --- | --- | --- |
|  |  | 1999 | 2001 | 2002 | 2005 | 2008 | | 2009 | | 2011 | 2014 | |
| All | |  |  |  |  |  | |  | |  |  | |
|  | RII (95% CI) ^a^ | 1.42 (1.00-2.01)* | 1.03 (0.79-1.36) | 1.51 (1.11-2.06)** | 1.63 (1.22-2.16)*** | 1.37 (1.09-1.74)** | | 1.65 (1.31-2.08)*** | | 1.68 (1.37-2.06)*** | 1.61 (1.31-1.98)*** | |
|  | SII (95% CI) ^a^ | 1.35 (-0.66-3.35) | 0.49 (-1.57-2.55) | 3.21 (1.02-5.39)** | 4.39 (2.41-6.38)*** | 3.13 (1.08-5.18)** | | 5.15 (3.03-7.27)*** | | 5.57 (3.52-7.63)*** | 5.08 (3.11-7.04)*** | |
| Female | |  |  |  |  |  | |  | |  |  | |
|  | RII (95% CI) ^b^ | 1.47 (0.90-2.38) | 1.39 (0.95-2.05) | 1.94 (1.26-3.01)** | 1.92 (1.26-2.94)** | 2.13 (1.49-3.04)*** | | 2.22 (1.56-3.16)*** | | 2.44 (1.82-3.26)*** | 1.82 (1.34-2.48)*** | |
|  | SII (95% CI) ^b^ | 1.95 (-1.07-4.96) | 2.70 (-0.25-5.66) | 5.65 (2.48-8.82)*** | 5.32 (2.37-8.26)*** | 6.81 (4.07-9.56)*** | | 7.50 (4.61-10.40)*** | | 9.79 (7.01-12.56)*** | 5.98 (3.37-8.59)*** | |
| Male | |  |  |  |  |  | |  | |  |  | |
|  | RII (95% CI) ^b^ | 1.34 (0.83-2.15) | 0.74 (0.50-1.08) | 1.13 (0.73-1.73) | 1.39 (0.95-2.04) | 0.92 (0.67-1.27) | | 1.25 (0.92-1.69) | | 1.14 (0.86-1.51) | 1.41 (1.07-1.85)* | |
|  | SII (95% CI) ^b^ | 1.12 (-1.50-3.73) | -1.21 (-3.88-1.47) | 0.14 (-3.01-3.30) | 2.01 (-0.65-4.68) | -0.47 (-3.54-2.60) | | 1.87 (-1.07-4.81) | | 1.18 (-1.96-4.32) | 3.16 (0.18-6.14)* | |
| ^a^ Age group, gender, marital status, household size, education fractional rank score, and household income fractional rank score were included | | | | | | | | | | | | |
| ^b^ Age group, marital status, household size, education fractional rank score, and household income fractional rank score were included | | | | | | | | | | | | |
|  | | | | | | | | | | | | |
